# Disruption of the alox5ap gene ameliorates focal ischemic stroke: possible consequence of impaired leukotriene biosynthesis

**DOI:** 10.1186/1471-2202-13-146

**Published:** 2012-11-30

**Authors:** Jakob O Ström, Tobias Strid, Sven Hammarström

**Affiliations:** 1 Division of Clinical Chemistry, Department of Clinical and Experimental Medicine, Faculty of Health Sciences, Linköping University, Linköping, Sweden; 2 Division of Cell Biology, Department of Clinical and Experimental Medicine, Faculty of Health Sciences, Linköping University, Linköping, Sweden

**Keywords:** Brain infarction, Leukotrienes, Alox5ap protein, FLAP, Mice, Middle cerebral artery occlusion

## Abstract

**Background:**

Leukotrienes are potent inflammatory mediators, which in a number of studies have been found to be associated with ischemic stroke pathology: gene variants affecting leukotriene synthesis, including the FLAP (ALOX5AP) gene, have in human studies shown correlation to stroke incidence, and animal studies have demonstrated protective properties of various leukotriene-disrupting drugs. However, no study has hitherto described a significant effect of a genetic manipulation of the leukotriene system on ischemic stroke. Therefore, we decided to compare the damage from focal cerebral ischemia between wild type and FLAP knockout mice. Damage was evaluated by infarct staining and a functional test after middle cerebral artery occlusion in 20 wild type and 20 knockout male mice.

**Results:**

Mortality-adjusted median infarct size was 18.4 (3.2-76.7) mm^3^ in the knockout group, compared to 72.0 (16.7-174.0) mm^3^ in the wild type group (p < 0.0005). There was also a tendency of improved functional score in the knockout group (p = 0.068). Analysis of bone marrow cells confirmed that knockout animals had lost their ability to form leukotrienes.

**Conclusions:**

Since the local inflammatory reaction after ischemic stroke is known to contribute to the brain tissue damage, the group difference seen in the current study could be a consequence of a milder inflammatory reaction in the knockout group. Our results add evidence to the notion that leukotrienes are important in ischemic stroke, and that blocked leukotriene production ameliorates cerebral damage.

## Background

Ischemic stroke is one of the leading causes of death and long-term disability in the world. Despite the allocation of huge research efforts in recent years, treatment options for ischemic stroke remain few. Therefore, investigations into the pathophysiological intricacies of the disease are crucial to provide new drug targets.

Inflammation is a prominent feature of stroke pathophysiology, and has attracted substantial research interest. The massive cell death in the infarct area triggers an acute and prolonged inflammatory process in the brain, characterized by activation of microglia, production of inflammatory cytokines and infiltration of various inflammatory cells, including neutrophils, T-cells and monocytes/macrophages, into the damaged tissue. Inflammation contributes to tissue damage, and especially the early inflammatory cell infiltration and cytokine production seem to be predominantly deleterious [1].

Mounting evidence indicates that leukotrienes (LTs), a group of potent inflammatory mediators [2], have an important role in cerebral ischemia, and that components involved in the LT cascade may be attractive drug targets. LTs are formed from arachidonic acid (AA) by 5-lipoxygenase (5-LO) upon immunological or inflammatory challenge. 5-LO activating protein (FLAP) is an integral membrane protein localized to the nuclear envelope and endoplasmic reticulum. Both 5-LO and FLAP are required for formation of LTA_4_ from endogenous AA [3-8], and LTA_4,_ is the precursor of all AA-derived effector LTs. The expression of 5-LO [9] and LT receptors [10-13] is affected by cerebral ischemia, and the importance of eicosanoids has been proposed from the observation that AA concentrations are highest in ischemic brain regions most sensitive to stroke [14]. Human genetic studies have demonstrated a significant correlation between stroke risk and polymorphisms in several of the LT-associated genes, e.g. ALOX5AP encoding FLAP [15-17], as well as genes encoding LTC_4_ synthase [16,18] and cysteinyl (Cys) LT receptors [16]. Also, a large Swedish epidemiologic study recently showed that intake of montelukast, a CysLT_1_R antagonist, was associated with a decreased risk of recurrent stroke [19]. Furthermore, animal studies have demonstrated that drugs interfering with components of the LT pathway, such as 5-LO [20-22] and LT receptors [23-28], ameliorate cerebral ischemic damage. Effects mediated by both of the established CysLT receptors, CysLT_1_R [13,24,26-28] and CysLT_2_R [10], appear to be involved in ischemic brain injury. Furthermore, the proposed CysLT receptor GPR17 was found to be up-regulated in damaged tissues, and knockout of the GPR17 gene reduced neuronal injury after ischemia [29,30].

The use of genetically modified animals is an important tool in the elucidation of biological mechanisms. However, in the case of LT effects on stroke, such studies remain scarce. The only one we are aware of investigated the effect of disrupting the 5-LO gene in mice, but no significant effects on focal cerebral ischemia were seen [31]. Because of the suggested importance of FLAP in human stroke studies, we decided to investigate the effect of FLAP gene knockout in a rodent middle cerebral artery occlusion (MCAo) model. The hypothesis was that the decreased LT production in the genetically modified mice would ameliorate the damage from ischemic stroke.

## Results

### FLAP knockout mice do not produce leukotrienes

**Figure 1:**
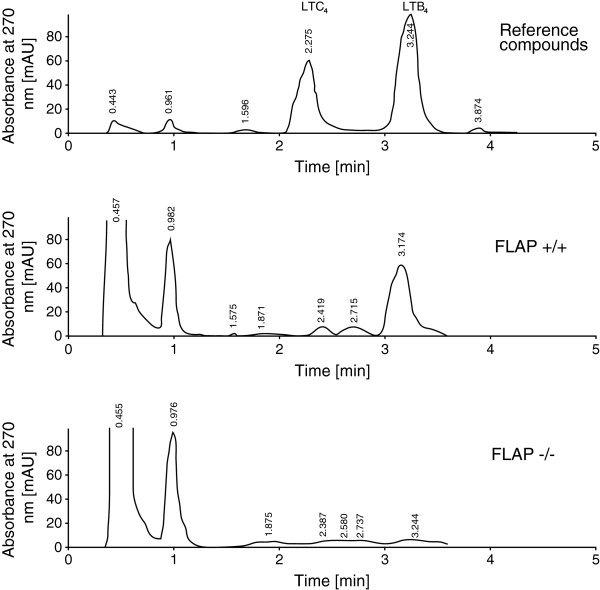
**Leukotriene formation was blocked in FLAP knockout mice**. Bone marrow cells obtained from wild type and FLAP knockout animals were incubated with 50 μM AA and 20 μM calcium ionophore A23187 for 20 min at 37°C. Reactions were stopped with ice cold methanol and supernatants were analyzed by RP-HPLC as described in Materials and Methods. The absorbance at 270 nm, corrected for stray absorbance at 360 nm, was recorded versus time. Retention times in minutes are indicated for each component in the chromatograms. *Upper panel*: analysis of synthetic LTB_4_ and LTC_4_. *Middle panel*: LTB_4_ was formed by bone marrow cells from wild type mice, but not from knockout mice (*lower panel*).

LTA_4_ is an obligatory intermediate in the biosynthesis of LTB_4_ and LTC_4_[32]. It is unstable and thus difficult to measure by direct methods. Therefore, we measured its downstream product LTB_4_ which is efficiently formed from LTA_4_ by the widely expressed enzyme LTA_4_ hydrolase. Bone marrow cells from wild type and knockout mice were incubated without cysteine with AA and calcium ionophore A23187. The lack of cysteine prevents formation of LTC_4_[33,34] whereas formation of LTB_4_ proceeds normally. RP-HPLC analyses showed that LTB_4_ was formed by wild type, but not by FLAP knockout, bone marrow cells (Figure 1).

### FLAP knockout decreased mortality-adjusted infarct size

**Figure 2:**
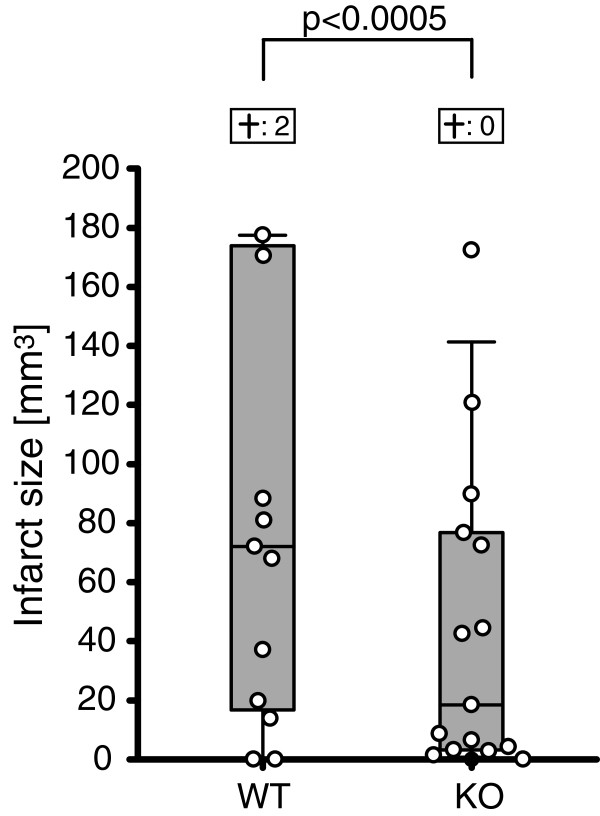
**Infarct sizes were decreased in FLAP knockout mice**. In terms of mortality-adjusted median infarct size, the knockout animals were protected from focal cerebral ischemia in comparison to their wild type counterparts (p < 0.0005). Infarct size is presented in mm^3^. The segments of the box plots depict the 10^th^, 25^th^, 50^th^, 75^th^ and 90^th^ percentile. The boxes above the box plots represent the mortality in the two groups. WT = Wild type group, KO = FLAP knockout group.

In the wild type group, two of the included animals died within 24 h after MCAo, while no included animals died in the FLAP knockout group. Mortality-adjusted median infarct size was 18.4 (3.2-76.7) mm^3^ in the knockout group, compared to 72.0 (16.7-174.0) mm^3^ in the wild type group (p < 0.0005; Figure 2).

### FLAP knockout had no significant effect on mortality-adjusted tail swing test performance

**Figure 3:**
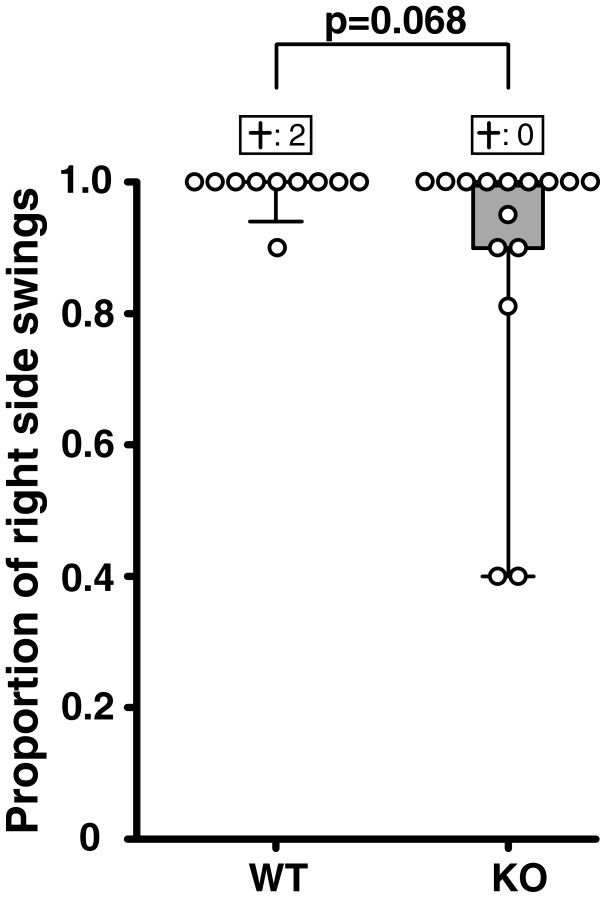
**Tendency of improved functional outcome in FLAP knockout mice**. No significant effect of the genotype difference was found in the mortality-adjusted tail swing test, although a trend of better performance in the knockout group was seen (p = 0.068). The proportion of right sided swings is plotted on the Y-axis. The segments of the box plots depict the 10^th^, 25^th^, 50^th^, 75^th^ and 90^th^ percentile. The boxes above the box plots represent the mortality in the two groups. WT = Wild type group, KO = FLAP knockout group.

Before MCAo, the wild type and knockout groups scored close to the theoretical baseline index of 0.50 (equal number of swings to the left and right), with tail swing indices (proportion of right-sided swings) of 0.50 (0.40-0.60) and 0.65 (0.53-0.70), respectively. The mice in both groups were severely affected by the ischemic stroke, and as much as 91% of the wild type and 60% of the knockout animals scored maximally (1.00 = 100% of swings to the right) in the tail swing test, thus decreasing the test sensitivity. Despite this, a trend of better outcome, which however did not reach statistical significance (p = 0.068), was seen in the knockout group. Mortality-adjusted median tail swing index (proportion of right side swings) was 1.00 (1.00-1.00) in the wild type group, compared to 1.00 (0.90-1.00) in the knockout group (Figure 3).

### Perioperative physiological monitoring

The physiological variables monitored during MCAo were similar between the two groups, as presented in Table 1, indicating that they responded similarly to the anesthesia.

**Table 1:** Perioperatively monitored physiological variables

**Group**	**O**_**2**_**Saturation [%]**	**Heart rate [bpm]**	**Pulse distention [μm]**	**Breath rate [bpm]**	**Breath distention [μm]**
Wild type	94.6 (91.1-97.4)	549.0 (523.9-568.6)	25.9 (18.2-36.3)	53.7 (44.7-76.1)	23.8 (19.0-33.1)
Knockout	95.2 (89.5-96.2)	531.5 (491.3-566.5)	17.7 (13.1-24.2)	57.9 (45.6-67.7)	20.6 (19.0-21.9)

All values are presented as median (1^st^ quartile, 3^rd^ quartile).

### Exclusions

In total, 7/20 (35%) animals in the wild type group and 5/20 (25%) animals in the knockout group were excluded. All exclusions were due to criterion #1; death during MCAo (Table 2).

**Table 2:** Number of animals excluded according to respective criteria in the two groups

**Group**	**Criterion #1: Death before the end of MCAo surgery [n]**	**Criterion #2: Failure to insert silicone coated filament at least 12 mm into the CCA [n]**	**Criterion #3: Signs of pathology prior to MCAo surgery [n]**
Wild type	7	0	0
Knockout	5	0	0

### Cerebrovascular anatomy

**Figure 4:**
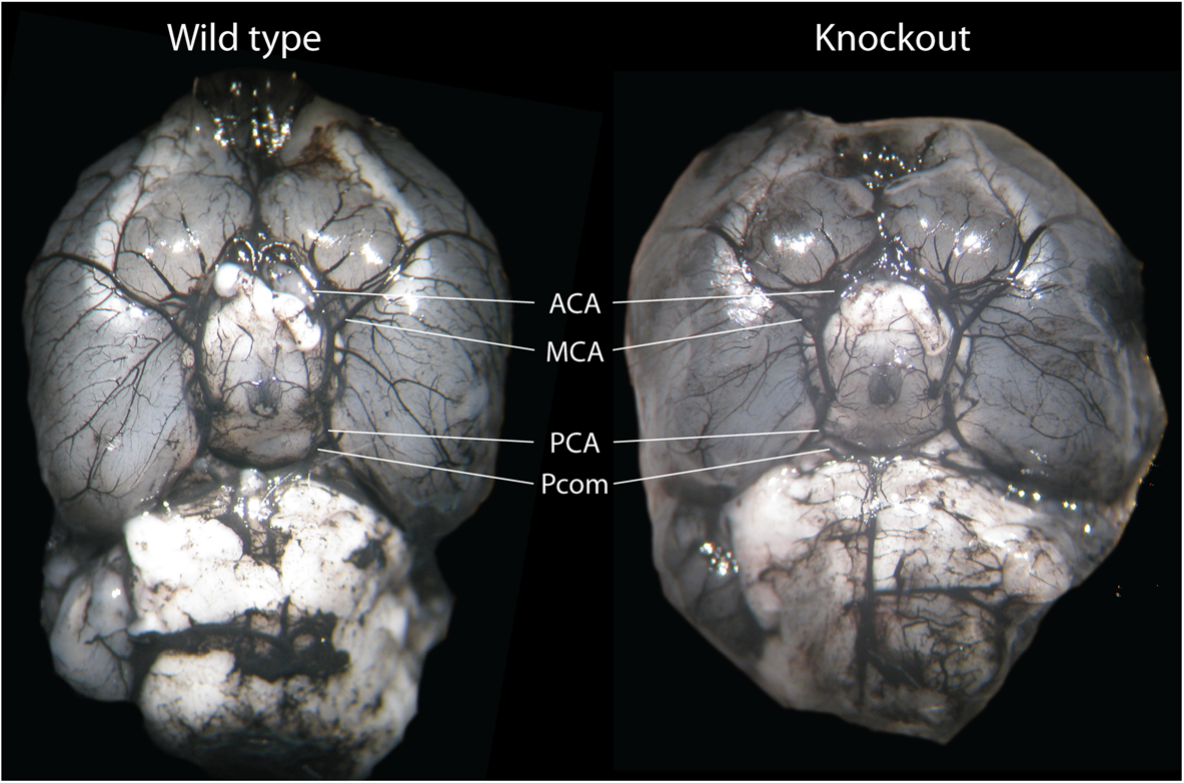
**Cerebrovascular anatomy in wild type and knockout mice**. No cerebrovascular differences between wild type and knockout animals were found. Brains from carbon black perfused wild type and FLAP knockout animals are depicted. Anterior cerebral artery (ACA), middle cerebral artery (MCA), posterior cerebral artery (PCA) and posterior communicating artery (Pcom) are pointed out in the figure.

Cerebrovascular anatomy was analyzed in 10 separate animals by carbon black perfusion and quantitative image analysis. The analyzed vessels (right and left MCA, anterior cerebral artery (ACA), posterior cerebral artery (PCA) and posterior communicating artery (Pcom)), were observed in all animals. Neither multiple T-tests nor two-way ANOVA (with the factors [genotype], p = 0.204, and [vessel]) revealed any significant differences between knockout and wild type animals regarding vessel outer diameter (Figure 4, Table 3).

**Table 3:** Vessel outer diameter in the two groups

**Vessel**	**Wild type [mm] (mean ± SD)**	**Knockout [mm] (mean ± SD)**	**T-test p-value**
MCA right	0.14 ± 0.012	0.16 ± 0.031	0.15
MCA left	0.14 ± 0.011	0.14 ± 0.015	0.73
ACA right	0.16 ± 0.041	0.17 ± 0.028	0.84
ACA left	0.17 ± 0.033	0.16 ± 0.031	0.61
PCA right	0.18 ± 0.030	0.18 ± 0.036	0.88
PCA left	0.17 ± 0.017	0.17 ± 0.027	0.63
Pcom right	0.12 ± 0.016	0.15 ± 0.033	0.07
Pcom left	0.13 ± 0.007	0.13 ± 0.022	0.88

## Discussion

Knockout of the FLAP gene was associated with ceased LT production and amelioration of stroke damage in terms of mortality-adjusted infarct size. Furthermore, there was a clear trend of improved mortality-adjusted functional test performance in the knockout group.

Activation of LT synthesis involves translocation to the nuclear envelope and endoplasmic reticulum of cytosolic phospholipase A_2_ (cPLA_2_) and of 5-LO from the cytosol and nuclear matrix. cPLA_2_ subsequently releases AA from membrane phospholipids [3,5,35]. FLAP resides at these locations as an integral membrane protein [3-6,8] and facilitates the transfer of AA from cPLA_2_ to 5-LO [36]. The labile intermediate LTA_4_, formed by 5-LO, is converted to LTB_4_ by LTA_4_ hydrolase or to LTC_4_ by LTC_4_ synthase. LTC_4_ is exported from the cell and metabolized to two other cysteinyl (Cys) LTs, LTD_4_ and LTE_4_. Both 5-LO and FLAP are required for LTA_4_ synthesis from endogenous AA [7], and both FLAP knockout mice and 5-LO knockout mice lack detectable LT production [37]. This was confirmed for the FLAP knockout mice in the current study by stimulating WT and KO bone marrow cells under conditions which allow LTB_4_ formation, but not LTC_4_ formation to occur due to lack of cysteine in the incubation mixture [33,34]. Thus LTB_4_ represents the total production of LTs from LTA_4_. LTB_4_ is a potent chemoattractant, which recruits inflammatory cells to sites of inflammation [38-40]. It also contributes to leukocyte accumulation by attenuation of leukocyte apoptosis [41,42]. CysLTs cause wide-spread plasma leakage by increasing vascular permeability and attract subsets of T-cells [43-45]. They also activate dendritic cells and their cytokine release [46,47] as well as mast cell cytokine production [48], which may also influence the inflammatory state. Hence, alterations in the LT pathway affect the inflammatory response, and such alterations could in turn have an impact on cerebral ischemia.

It should be noted, however, that inflammation is not only an important feature in the specific infarct process, but also in the pathology of atherosclerosis. As mentioned above, several genetic studies on human populations have linked LT-related genes to altered stroke incidence [15-18]. Such studies, however, do not confer firm evidence regarding the mechanisms of the effects. This emphasizes the importance of animal studies, since they are *sin qua non* for investigating biological mechanisms. As already mentioned, several reports, using models similar to those in the current study, have demonstrated protection against stroke by drugs blocking LT effects, such as montelukast [23,24] and pranlukast [27,28], strongly suggesting LT-related effects on the specific infarct pathophysiology. Experiments using drugs and those employing genetically modified animals are important complements to each other. The study using a 5-LO knockout mouse strain, mentioned above, showed no effects on infarct size after transient MCAo. That study, however, only included 6 mice per transient MCAo group, and with infarct size coefficient of variation (standard deviation divided by mean value) of around 42% and α = 0.05, the chance (statistical power) of detecting for example a 30% difference was 45.1%. In other words, the study was underpowered in this specific respect, thus not substantiating negative conclusions [31]. To the best of our knowledge, the current study is the first to show that a genetic distortion of the LT system ameliorates the detrimental effects of cerebral ischemia.

It should be noted that altered inflammatory response is not the only possible mechanism for the decreased infarct volumes in the knockout group in the current experiment. Even though the cerebrovascular anatomy was similar between the groups, the lack of leukotriene production may theoretically have affected the blood flow to the brain, which in turn could decrease the infarct size. For example, exogenous LTD_4_ increases the blood pressure in rats [49] and the FLAP inhibitor MK886 ameliorates hypertension in L-NAME treated rats [50].

It is a well-known problem that MCAo studies often suffer from high random variability regarding infarct size, and numerous attempts have been made to address this [51-54]. The source of this variability can be a consequence of for example inconsistency in the filament insertion procedure and to subtle, individual variations in cerebral vasculature as well as in peroperative hydration status and body temperature. In the current study, efforts to minimize random variability included a strictly standardized operation procedure performed by one single surgeon, peroperative surveillance of physiological parameters and the use of an inbred mouse strain, minimizing inter-individual differences.

### Strengths and weaknesses of the current study

A frequent problem in animal stroke studies is that mortality is neglected, and not included in the final analysis. A strength of the current study is that this was addressed by combining mortality with infarct size and functional score, respectively, in two mortality-adjusted non-parametrical models. The advantage of this approach is that the importance of the extreme outcome of death is acknowledged. A theoretical drawback is that if mortality was very high in one of the groups, that factor itself could contribute with so much group difference that any other variable combined with the mortality rate would seem significant. In the current study, with only 2 included cases of death, this was not a concern.

Even though 2,3,5-triphenyltetrazolium chloride (TTC) staining is a well-used and validated method for infarct size assessment, other staining procedures could have provided differentiated information regarding the mode of cell death. It should be noted that any eventual differences between the groups regarding mode of cell death remain undisclosed in the current experimental setup. Such differences may potentially contribute to explaining the mechanism of the reduced infarct sizes in the knockout group, and merit attention in future studies.

In a preceding pilot study, different MCA occlusion times were tested with the result that for this specific mouse type, 120 minutes was needed to ensure a relatively consistent infarction. This however caused quite high mortality if longer convalescence periods were adopted, which was why we settled for 24 h even though longer survival times could be beneficial. Theoretically, the infarct evolution may merely have been delayed in the knockout group.

## Conclusions and future perspectives

We conclude that disruption of the FLAP gene decreases mortality-adjusted infarct size following MCAo in mice. It merits emphasis that we do not draw conclusions regarding the effects on infarct sizes or mortality in separate, but regarding a combination of the two, which in our opinion is more relevant. An interesting avenue for future research would be to study effects of combinations of genetic modifications and LT inhibiting drugs, to be able to further elucidate the exact mechanisms involved. Future studies using the FLAP knockout model on other kinds of brain inflammation might provide important new information concerning the pathogenesis of such diseases.

## Methods

### Animals

A FLAP-knockout mouse strain [37], generously provided as frozen embryos by Dr. Beverly H. Koller, was bred at an animal facility in Linköping, Sweden. The animals were housed in 12 h/12 h light/dark cycles (lights on at 7 AM). Food (801730, Special Diets Service, Essex, England) and tap water were provided *ad libitum*. The strain was maintained by breeding against 129SvEv mice (Taconic, Tornbjerg, Denmark) and 5 generations of backcrossing were made before the stroke experiments. All procedures were conducted in accordance with the National Committee for Animal Research in Sweden and Principles of Laboratory Animal Care (NIH publication no. 86–23, revised 1985). The protocol was approved by the Local Ethics Committee for Animal Care and Use in Linköping.

### Experimental procedures

Forty male mice (age: 126 (94–151) days, weight: 28.4 (27.8-29) g) were used for MCAo. Twenty homozygous FLAP^−/−^ mice were consecutively selected for the knockout group, while 20 consecutively selected FLAP^+/+^ mice served as controls. The experimenter responsible for performing the MCAo and infarct assessments (JOS) was blinded to the genotype of all mice from the start of experiments and until after infarct size analyses. The mice were operated in random order.

MCAo was performed using the intraluminal filament method [55,56]. The mice were anesthetized with isoflurane (4.5% for induction, 1.8% for maintenance; Forene®, Abbott Scandinavia AB, Solna, Sweden) in an oxygen/nitrous oxide 30%/70% mixture, and laid in supine position on a thermostatic heating pad connected to an anal thermometer (50–7061, Harvard Apparatus, Holliston, MA, USA). Eye gel (Lubrithal™, VetXX, Uldum, Denmark) was utilized to protect the eyes during anesthesia, and before surgery, animals received 1 mL saline subcutaneously for fluid replenishment. The throat of the mouse was shaved and washed with Iodopax (Jodopax vet®; Pharmaxim AB, Helsingborg, Sweden) prior to incision. During MCAo anesthesia, O_2_ saturation, heart rate, pulse distention, breath rate and breath distention were monitored by pulse oximetry (SLS-MO-00404, MouseOx, Allison Park, PA, USA). A 2 cm midline incision was made over the trachea, and the left common (CCA), external (ECA) and internal carotid arteries (ICA) were freed from surrounding tissue. After ligating (6–0 silk suture, Johnson & Johnson, New Brunswick, NJ, USA) the left CCA and ECA, a suture was prepared around the left ICA, and the ICA was temporarily clipped (8 mm artery clip, Rebstock Instruments Gmbh, Dürbheim, Germany). A small incision was subsequently made in CCA, just proximal to the bifurcation, and a silicon-coated filament (502756, Doccol, Redlands, CA, USA) was inserted until a slight resistance indicated correct placement. The intraluminal filament was secured by a knot, the wound was closed by sutures, and the mouse was allowed to wake up. After two hours of occlusion, the animal was reanesthetized, the filament withdrawn, and the wound closed anew. Topical lidocain gel (Xylocain 2%, AstraZeneca AB, Södertälje, Sweden) was used for postoperative analgesia.

#### Postoperative care

After surgery, the mice were housed solitarily, without nesting material, in a heated (25-26°C) environment until sacrifice 24 h later. Food pellets, soaked in water, were placed on a Petri dish at the cage floor to promote eating.

#### Physiological testing

Before MCAo, and one day postoperatively, the mice were tested for right-left asymmetry by means of the tail swing test [57]. The animal was held in the tail above the cage, and the directions of the first 20 lateral attempts to reach the experimenter’s hand holding the tail were recorded. A right-left index was subsequently calculated by dividing the number of right side swings by the total number of swings. An index of 0.5 therefore means that the animal swinged an equal number of times to the right as to the left.

#### Lesion measurements

The animals were lightly anesthetized in isoflurane 24 h after MCAo and decapitated in a small animal guillotine. The brains were dissected, immersed in cold water for two minutes, and cut in 1 mm slices in a mouse brain matrix (BSMAS001-1, Zivic Instruments, Pittsburgh, PA, USA). The slices were soaked in TTC (Sigma-Aldrich, CAS# 298-96-4) in PBS (pH 7.4) for 15 minutes in a small Petri dish maintained at 37°C. The slices were scanned (ScanJet 2c, Hewlett-Packard) and infarct areas were calculated as described by Goldlust [58], using an automatic 40% green spectrum threshold (SigmaScan Pro version 5, Systat Software Inc., San Jose, CA, USA). When infarct areas in each slice had been established, the lesion volume was calculated by multiplying the average infarct area of two adjacent slices with the thickness of the tissue in between, which was then summed up to a total infarct size. Infarct volume is expressed as mm^3^, and corrected for edema with the assumption that the edema in the infarcted hemisphere is not restricted to the infarct, but also affects surrounding tissue.

### Exclusion criteria

Exclusion criteria were established prior to the start of the experiments:

1. Death before the end of MCAo surgery

2. Failure to insert silicone coated filament at least 12 mm into the CCA

3. Signs of pathology prior to MCAo surgery

### Analysis of cerebrovascular anatomy

For analysis of cerebrovascular anatomy, 5 knockout and 5 control mice were perfused with carbon black ink (Schribtol, drawing ink for calligraphy, Pelikan, Hannover, Germany) into the left heart ventricle. The brain was subsequently dissected, and care was taken not to damage the vasculature. The ventral aspect of the brain was photographed (Ixus 85 IS, Canon, Tokyo, Japan) through an operating microscope. The outer diameters of the right and left MCA, ACA, PCA and Pcom were measured using Sigmascan.

### Analysis of leukotriene production

Bone marrow cells were collected from femurs and tibias of euthanized knockout (FLAP^−/−^) and control (FLAP^+/+^) mice. Equal numbers of cells were incubated for 20 min at 37°C in PBS containing 50 μM AA and 20 μM calcium ionophore A23187. Reactions were stopped by addition of ice-cold methanol. Supernatants were collected, cleared from cell debris, evaporated and the residues were dissolved in methanol and analyzed by RP-HPLC using a Poroshell 120 EC-C_18_ column (particle size 2.7 μm, internal diameter 3 mm, length 50 mm), a 1200 series HPLC, and a model 1290 diode array detector (all from Agilent Technologies). LTs were eluted using methanol/water 7:3 (v/v) plus 0.1% acetic acid, adjusted to pH 5.7 by ammonium hydroxide (phase A) for 3 min followed by a linear gradient up to 30% methanol in phase A for 5 min, and finally a linear gradient up to 100% methanol for 1 min, all at a flow rate of 0.5 ml per min.

### Statistics

For the infarct experiments, an a priori power calculation based on an expected group difference of 40% and an expected coefficient of variation of 40%, yielded (1-β) = 0.869 for n = 20 mice in each group. Mortality after MCAo was combined with lesion size and tail swing test performance, respectively, in a non-parametric model in which death was considered the worst possible outcome. Wilcoxon’s rank-sum test, with α = 0.05, was used for these analyses (Systat version 11, Systat Software, Inc. CA, USA). Infarct and test performance data are presented as median (1^st^ quartile-3^rd^ quartile) throughout.

Cerebrovascular anatomy was compared by t-tests for each vessel, and by two-way ANOVA with the factors [genotype] and [vessel] (SPSS, Version 20, IBM Corporation, Armonk, NY, USA). For the t-tests, N = 5 in each group rendered an observed power of 0.816 to discover a 30% vessel diameter difference between the groups, given an α = 0.05 (Systat version 11, Systat Software, Inc. CA, USA). Vessel diameter data are presented as mean ± standard deviation throughout.

### ARRIVE and STAIR

The experiment design and manuscript conform to the ARRIVE-guidelines of 2011 [59]. Of the 8 STAIR-criteria [60], developed for preclinical stroke experiments, 4 (extensive physiological monitoring, randomization and blinding, more than one effect measure, [intention to] publish in a peer-review journal) were fulfilled.

## Competing interests

The authors declare that there are no competing interests.

## Authors’ contributions

JOS contributed to designing the study, performed the infarct surgeries, outcome assessments, infarct result analyses and drafted the manuscript. TS contributed to designing the study, handled the breeding, leukotriene analyses and revised the manuscript. SH conceived and contributed to designing the study and revised the manuscript. All authors read and approved the final manuscript version before submission.
